# A Role for *LHC1* in Higher Order Structure and Complement Binding of the *Cryptococcus neoformans* Capsule

**DOI:** 10.1371/journal.ppat.1004037

**Published:** 2014-05-01

**Authors:** Yoon-Dong Park, Soowan Shin, John Panepinto, Jeanie Ramos, Jin Qiu, Susana Frases, Patricia Albuquerque, Radames J. B. Cordero, Nannan Zhang, Uwe Himmelreich, David Beenhouwer, John E. Bennett, Arturo Casadevall, Peter R. Williamson

**Affiliations:** 1 Laboratory of Clinical Infectious Diseases, National Institute of Allergy and Infectious Diseases, National Institutes of Health, Bethesda, Maryland, United States of America; 2 Section of Infectious Diseases, Department of Medicine, University of Illinois at Chicago College of Medicine, Chicago, Illinois, United States of America; 3 Department of Microbiology and Immunology, University at Buffalo, the State University of New York, Buffalo, New York, United States of America; 4 Department of Microbiology and Immunology and Division of Infectious Diseases of the Department of Medicine, Albert Einstein College of Medicine, Bronx, New York, New York, United States of America; 5 Laboratorio de Ultraestrutura Cellular Hertha Meyer, Instituto de Biofisica Carlos Chagas Filho, Universidade Federal do Rio de Janeiro, Rio de Janeiro, Brazil; 6 Biomedical NMR Unit, Department of Medical Diagnostic Sciences, Division of Radiology, Katholieke Universiteit Leuven, Leuven, Belgium; 7 Division of Infectious Diseases, Veterans Affairs Greater Los Angeles Healthcare System, Los Angeles, California, United States of America; 8 Department of Medicine, David Geffen School of Medicine, University of California, Los Angeles, California, United States of America; University of Birmingham, United Kingdom

## Abstract

Polysaccharide capsules are important virulence factors for many microbial pathogens including the opportunistic fungus *Cryptococcus neoformans*. In the present study, we demonstrate an unusual role for a secreted lactonohydrolase of *C. neoformans*, *LHC1* in capsular higher order structure. Analysis of extracted capsular polysaccharide from wild-type and *lhc1*Δ strains by dynamic and static light scattering suggested a role for the *LHC1* locus in altering the capsular polysaccharide, both reducing dimensions and altering its branching, density and solvation. These changes in the capsular structure resulted in *LHC1*-dependent alterations of antibody binding patterns, reductions in human and mouse complement binding and phagocytosis by the macrophage-like cell line J774, as well as increased virulence in mice. These findings identify a unique molecular mechanism for tertiary structural changes in a microbial capsule, facilitating immune evasion and virulence of a fungal pathogen.

## Author Summary

Polysaccharide capsules are important virulence factors in pathogenic microbes that provide a protective coat against host immunity. *Cryptococcus neoformans* is a pathogenic encapsulated yeast that is a major opportunistic infection, causing approximately 600,000 cases of meningitis per year in AIDS patients globally, and whose polysaccharide capsule is a major virulence factor. While extensive work has detailed the chemical components forming the cryptococcal capsule, the molecular events leading to the higher order assembly of the capsule, and its consequences on immune subterfuge remain unknown. In the present studies we used a proteomics method to identify a novel hydrolytic enzyme, lactonohydrolase (Lhc1) and used a variety of biophysical methods including dynamic and static light scattering as well as motility studies to show that extracted capsular polysaccharide undergoes remodeling in a *LHC1*-dependent fashion. This results in a more tightly compacted capsular structure that alters binding of anti-capsular antibodies and reduces binding by both human as well as mouse serum complement. Furthermore, *LHC1*-dependent capsular alterations serve to increase the virulence of the fungus in a mouse model, suggesting a novel role for this class of enzyme in capsular remodeling and immune evasion in microbial pathogenesis.

## Introduction

Polysaccharide capsules (PC) are highly diverse hydrated structures that provide microbes with a key defense against the host immune system [1]. For example, bacterial capsules confer resistance to complement-mediated opsonophagocytosis [2] and are an important property of highly virulent bacteria such as *Neisseria meningitidis* [3]. Among fungal pathogens, a prominent virulence factor of the opportunistic pathogen *Cryptococcus neoformans* is a large polysaccharide capsule with potent anti-phagocytic properties [4]. *C. neoformans* is a common cause of meningitis in parts of Africa [5], accounting for approximately 600,000 deaths annually [6].

The cryptococcal capsule is a hydrated polysaccharide gel, constituted by high-molecular weight polysaccharide polymers such as glucuronoxylomannan (GXM) which represents almost 90% of the total capsule with the remainder being glucuronoxylomannanogalactan (GXMGal) [7]. GXM is composed of a large backbone of 6-*O*-acetylated α-1,3-mannose residues with β-D-xylopyranosyl, β-D-glucuronosyl monosubstituted side chains [8]. Extensive work by numerous investigators has provided key insights into synthesis and virulence role of the capsular primary structure [7]. However, genes controlling or regulating higher order structures of the capsular polysaccharide have not been identified. This has been in part due to difficulties in assessing the tertiary structure of the cryptococcal polysaccharide.

Thus, to identify genes that may control the higher order organization of the capsular structure, we used a focused proteomic approach to identify capsular-associated proteins that may participate in remodeling of the cryptococcal capsule. Since current models suggest that the primary structure is synthesized within the cell cytoplasm [9], the hypothesis was that secreted proteins might be more likely to be involved in capsular tertiary structure. This approach identified a capsular lactonohydrolase of *C. neoformans* and a targeted mutant strain demonstrated a larger capsule size that was more permeable to dextran particles in a mutant strain defective in this hydrolytic activity. Recently applied biophysical methods [10] were then used to demonstrate that the mutant polysaccharide (PS) was larger, more hydrated and branched, evidenced by altered capsule nuclear magnetic spectra, zeta potential and polysaccharide hydrodynamic dimensions. The mutant also displayed an increase in antibody and serum-dependent phagocytosis by the macrophage cell line J774.16 cells, an increase in serum complement binding and reduced virulence in mice that could be reversed by depletion of complement using cobra-venom. These data thus identify *LHC1* as a unique example of a gene locus involved in modification of higher order capsular structure of a microbial pathogen and its role in immune evasion.

## Results

### Isolation of capsular-associated proteins from *C. neoformans* by a focused proteomic approach

**Figure 1:**
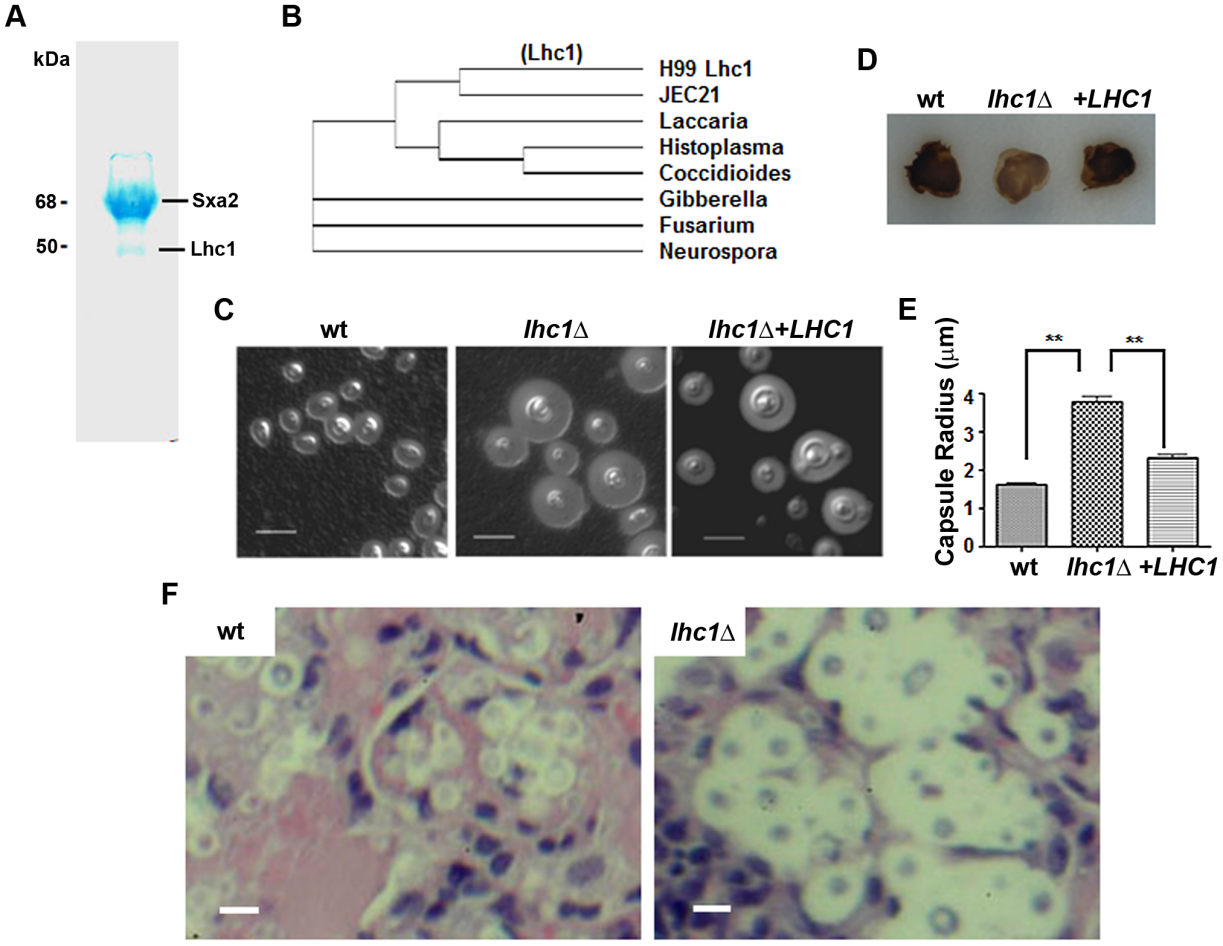
Identification of a capsular-adherent putative lactonohydrolase from *C. neoformans* and role in virulence-related phenotypes. (A) SDS-PAGE of DMSO-solubilized capsular proteins adsorbed on DEAE-agarose. (B) Clustal-W comparison of proteins sequences of closest matches of the 50 kDa Lhc1 sequence. Indicated strains were assayed for (D) laccase by melanin formation and, (C) capsule by India Ink microscopy. (E) Capsule radius of India ink-stained cells was determined in 100 cells of the indicated strains. (F) Capsule of *LHC1* strains during brain infection. Indicated strains (1×10^6^) were inoculated intravenously and when moribund, mice were sacrificed and brains excised, sectioned and stained with H&E as described in methods. Bar = 5 microns.

After extensive washing of cells, dimethyl sulfoxide (DMSO) was used to solubilize and remove the outer layers of the cryptococcal capsule without breakage of the cell wall as described previously [11]. Strain B-3501 was used because its smaller capsule produced relatively less capsular polysaccharide that could complicate protein purification. Interestingly, after recovery of crude protein from dialyzed DMSO-solubilized material by adsorption on diethylaminoethanol-agarose, only two prominent bands were identified on Coomassie-blue stained PAGE gels (Fig. 1A). Protein sequencing identified three cryptococcal proteins (see supplemental Table S1 in Text S1), each matching protein sequence within the serotype D (www.ncbi.nih.gov) as well as the H99 serotype A database (www.broad.mit.edu), indicating their presence in two strains representative of two important serotypes capable of causing human disease. The small number of protein bands was remarkable, considering the large number of secreted proteins of *C. neoformans* [12] and may be due to the presence of only a small population of capsular-associated proteins or to incomplete adsorption of proteins from solubilized capsule material by the DEAE-agarose matrix. Analysis of the CNAG_04753 amino acid sequence from the higher mobility band showed strong homology to a number of fungal lactonohydrolases including that from *Fusarium oxysporum* (E = e-119; Fig. 1B) and contained three conserved domains for this class of hydrolytic enzymes [13]. Interestingly, using the PROCARB carbohydrate binding prediction tool based on a database of known and modeled carbohydrate-binding protein structures [14], three putative amino acids were identified that could represent amino acids involved in such binding,W28, N454, and R456—all aromatic amino acids that have the capacity to form Pi(π) bond complexes with hexose sugars, a common mechanism of lectin binding to carbohydrates [15]. Sequence analysis of the lower mobility band (68 kDa) identified a mixture of a conserved hypothetical protein and a protein showing closest homology to Kex1 of yeast. Since these latter two proteins were less likely to be involved in capsular modifications, they were not analyzed further.

### Analysis of the role of *LHC1* in virulence-associated phenotypes of *C. neoformans*

A deletion strain was created in serotype A strain H99 to help identify a role for the putative lactonohydrolase from *C. neoformans*, Lhc1 using a strain of the serotype that is most predominant in human infections, serotype A [16]. As shown in Fig. 1C, a large increase in the size of the capsule was observed in the *lhc1Δ* mutant strain by India Ink microscopy grown in the presence of CO_2_, which was restored to approximately that of wild-type (wt) after complementation by a 3.6-kb fragment of the *LHC1* gene. Larger capsule was also evident in YPD after a 1 day incubation that showed poor capsule induction in the wt strain or after capsule induction in ASN minimal media, 1∶10 Sabouraud or RPMI media (Fig. S1 in Text S1). In contrast, deletion of *LHC1* had only a minor effect on other virulence factors such as laccase, measured by melanin formation (Fig. 1D) and no effect on urease activity or growth in YPD at 37°C (data not shown). Analysis of capsular radius of *lhcΔ* mutant cells using India ink microscopy induced by growth in the presence of 5% CO_2_ (Fig. 1E; p<0.01), ASN minimal media, 1∶10 SAB or RPMI demonstrated a significantly increased capsular radius compared to either the wt or complemented strains (Fig. S1 in Text S1; p<0.05). Interestingly, large capsules were also expressed by the *lhc1Δ* mutant in mouse brains (Fig. 1F, right panel) compared to that of wt (left panel) or the complemented strain (data not shown). These data establish a role for *LHC1* in the wt capsular phenotype both in vitro and in vivo.

### *LHC1* expresses a capsular cryptococcal lactonohydrolase

**Figure 2:**
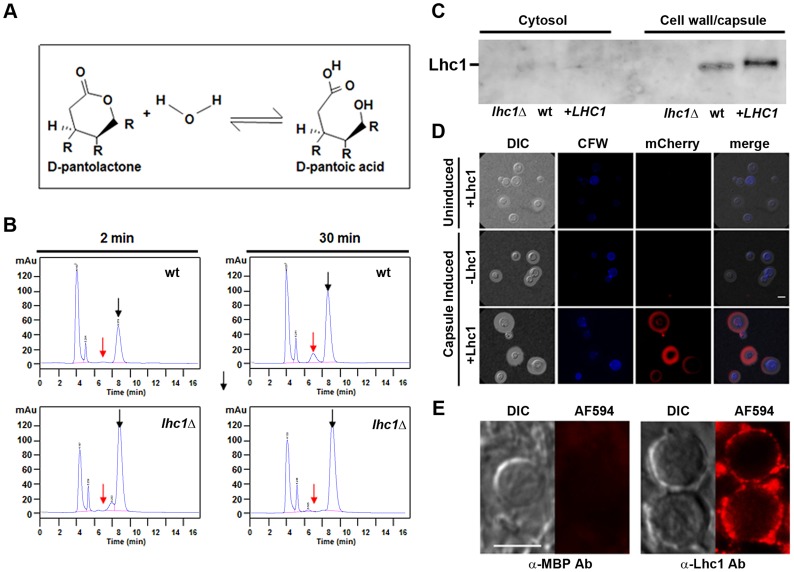
*C. neoformans* possesses a *LHC1*-dependent lactonohydrolase activity that is localized to the capsule and expressed during human infection. (A) Scheme of lactonohydrolase-dependent hydrolysis of D-pantolactone. (B) Indicated cells were grown in asparagine media (0.2% glucose) for 3 days, centrifuged and washed 3× in 10 mM Tris buffer, pH 7.0, incubated in the presence of 100 mM D-pantolactone at 37°C for the indicated times and the reaction terminated with methanol. Aliquots were then assayed for lactone (black arrow) or the hydrolyzed acid (red arrow) as determined using chemical standards. (C) Western blot analysis of polyclonal anti-Lhc1 reactivity against *C. neoformans* protein extracts. Protein supernatants of cell lysates (cytosol) or detergent-solubilized cell pellets (cell wall/capsule) from the indicated strains were prepared and western blot performed using polyclonal serum against the Lhc1 protein as described in Materials and Methods. (D) A *C. neoformans lhc1* **Δ** strain complemented with a vector expressing Lhc1-mCherry (+Lhc1) or empty vector alone (−Lhc1) was grown in YPD (uninduced) or induced on 1∶10 SAB (capsule induced) as described in Materials and Methods. (E) Sections of a brain autopsy specimen were obtained from a 30 y.o. female who died of severe and diffuse *C. neoformans* infection and stained with a mouse affinity-purified antibody against a maltose-binding protein-Lhc1 fusion protein (α-Lhc1 Ab) or anti-MBP polyclonal serum (α-MBP Ab) followed by incubation with a secondary Alexa Fluor 594 goat anti-rabbit IgG (AF494) according to Materials and Methods. Panels represent fluorescent antibody treated (AF594) or differential interference contrast microscopy (DIC) images. Cells were washed and mounted in anti-fade medium and imaged in an epifluorescence microscope using a 63× 1.4 NA objective. The exposure conditions were identical for each sample and are representative of 25 cells visualized. Bar = 5 microns.

To confirm the identity of *LHC1*, we assayed for hydrolysis of the aliphatic lactone, D-pantolactone (Fig. 2A) using a previously-described high performance liquid chromatographic method [17] after growth of wt and *lhc1Δ* mutant cells in minimal media for 3 days 30°C. As shown in Fig. 2B, wt fungal cells converted approximately 11.3+/−2.6% (SEM, N = 3) of the D-pantolactone to the corresponding acid in 30 min, whereas no significant hydrolysis was evident in the *lhc1*Δ mutant. A small shoulder on the substrate peak of the mutant reaction could represent an unknown breakdown product. However, we were not able to detect hydrolysis of the aromatic substrate 3,4-dihydrocoumarin by changes in UV absorption using a previously described method [18], suggesting a restriction to aliphatic lactones that might be expected within the polysaccharide matrix (data not shown). Recombinant Lhc1 was inactive in both assays which may be due to cryptococcal specific conformational modifications or a requirement for a specific carbohydrate binding cofactor for activity.

Further studies sought to confirm a capsular localization of Lhc1 suggested by DMSO-solubilization from intact cells. Western blots using mouse antiserum developed against a recombinant maltose-binding protein (MBP)-tagged Lhc1 fusion protein demonstrated an immunoreactive band from wt or *LHC1* complemented, but not *lhc1Δ* mutant strains of the appropriate molecular mass from SDS extracts of pelleted fractions enriched in cell wall/capsule but not cell lysis supernatants enriched in cytosolic proteins after homogenization (Fig. 2C). Lhc1-immunoreactivity was also not detected from culture supernatants or 20× concentrated culture supernatants (data not shown), further suggesting that Lhc1 was a capsular-associated protein. Control antibody raised against MBP alone showed no cross-reactivity against *C. neoformans* cellular materials by western blot as described (Fig. S5 in Text S1) [19].

In addition, Lhc1 was expressed under its native promoter as a *C. neoformans* codon-optimized mCherry fusion protein (Fig. 2D), which suggested that 1) Lhc1 expression is repressed under nutrient-rich conditions where capsule is repressed and 2) is successfully expressed and localized to capsule under conditions where capsule is induced, including 1∶10 SAB (Fig. 2D), ASN minimal media or RPMI (Fig. S2 in Text S1). Regulation appeared to be at the transcriptional level as quantitative RT-PCR studies demonstrated induction under capsule inducing conditions in either ASN minimal media, 1∶10 SAB or RPMI media after 24 h incubation, which was present for both the serotype A H99 strain as well as the serotype D strain (B-3501) used to identify the Lhc1 protein (Fig. S3 in Text S1). Additional studies utilized fluorescence immune-microscopy of sectioned *C. neoformans* cells to demonstrate capsular reactivity in a human autopsy specimen of a 30 year old female who died of overwhelming *C. neoformans* meningoencephalitis (Fig. 2E). These data suggest a role for Lhc1 expression in human infections. In summary, lactonohydrolase expressed from *C. neoformans* was localized to the capsular matrix of the fungus, although removal with detergent and DMSO as well as migration within an SDS-PAGE gel matrix suggested a non-covalent interaction.

### Role of *LHC1* in the capsular structure determined by Nuclear Magnetic Resonance (NMR)

**Figure 3:**
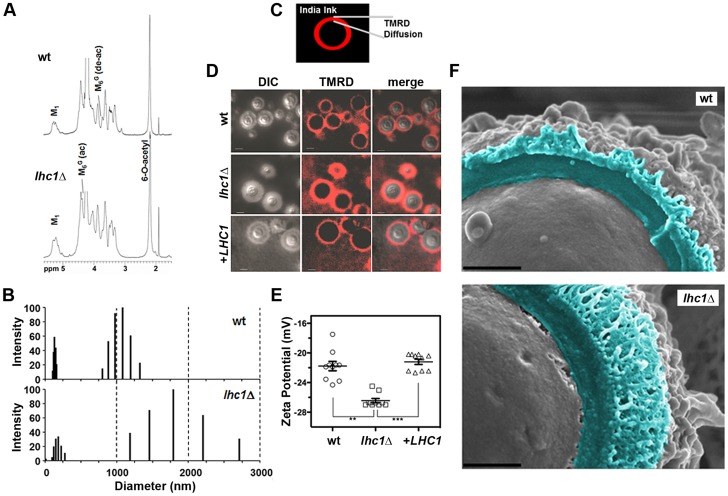
Deletion of *LHC1* is associated with alterations in higher order polysaccharide structure of solubilized polysaccharide. (A) Nuclear magnetic resonance (NMR) analysis of *C. neoformans* GXM. Isolated GXMs from indicated strains were dissolved in 0.5 ml of 99.96% D_2_O (Sigma, St. Louis, Mo.). NMR spectra were acquired with a Bruker Avance 600 NMR spectrometer, using a 5-mm (^1^H, ^13^C) inverse-detection dual-frequency probe, operating at 600.13 and 150.913 MHz, respectively. (B) TMRD diffusion was measured as the distance between the India Ink-capsule outer interface and the furthest region of diffusion of the fluorescent TMR-dextran. (C) TMRD diffusion: Fungal cells were induced for capsule, and treated with TMR-Dextran and India ink as described in Materials and Methods and observed for epifluorescence. Bar = 5 microns. (D) Capsule from the indicated strains was extracted with DMSO and polydispersity measured by Quasi elastic light scattering in a 90 Plus/Bi-MAS multi angle particle sizing analyzer as described in Materials and Methods. (E) Zeta potentials of capsular polysaccharide solutions from indicated strains at 1 mg/ml. Error bars represent SEM from 10 repeated measurements. (** p<0.01; *** p<0.001). (F) Fungal cells were subjected to cryo-scanning electron microscopy as described in Materials and Methods. Light blue coloring indicates outer region of cell wall and capsular structure exterior to the plasma membrane fracture plane. Bar = 500 nm.

Because of the larger capsule size of the *lhc1*Δ mutant evident on India ink staining, additional studies were conducted to assess alterations in the chemical structure of the capsule. Neutral sugar analysis did not identify large changes in substituent sugars although some increases in xylose and glucuronic acid as well as reductions in glucose were noted (Table S2 in Text S1). NMR spectroscopy of isolated soluble GXM from wt and the *lhc1*Δ strain also showed subtle differences in the GXM spectra that suggested alterations in higher order structure of the polysaccharide (Fig. 3A). The acetylation ratio was almost identical to the wt; however, mannosyl residues substituted with glucuronic acid residues were more frequently also 6-*O*-acetylated in the mutant (M_6_^G^-ac in Fig. 3A) in contrast to the deacetylated residues in the wt (M_6_^G^-deac). The ratio of mannose: xylose: glucuronic acid residues was 3∶2.1∶1, similar to wt. However, the exact chemistry of the cross-linkage of GXM chains was difficult to determine for the *lhc1*Δ strain. Samples are usually prepared for NMR spectroscopy by sonication to break-up large polysaccharides into smaller repeating units to reduce relaxation times and hereby allow for 2D NMR correlation spectroscopy [20]. However, conventional preparation in this case did not result in sufficiently small polysaccharide fragments to yield usable 2D NMR data for the determination of the smallest repeating carbohydrate structure in the *lhc1*Δ strain. High-power sonication resulted in complete break-up into monosaccharide units, suggesting that the larger polysaccharide fragments of the mutant exhibited higher levels of structural complexity in the form of branching or intermolecular cross-links that required higher sonication energies.

### Alteration of capsule permeability to dextran after deletion of *LHC1*

Because NMR spectroscopy suggested alterations in the higher order structure of the capsule, permeability was assessed using a fluorescent-labeled dextran dye (2,000 kDa) used previously in this organism [21]. India ink was added to the dextran suspension to allow demarcation of the exterior surface of the capsule. Full capsule thickness was determined as the distance between the fungal cell wall and the outer edge of the India ink exclusion zone (Fig. 3C). Comparison of the three strains demonstrated increased permeability of the *lhc1*Δ mutant versus that of either the wt or the complemented strain, as determined by the width of the zones of red fluorescence (wt: 1.38±0.07; *lhc1*Δ: 2.41±0.06; *lhc1*Δ+*LHC1*: 1.53±0.12; p<0.0001 for *lhc1*Δ versus either wt or complemented strain-Fig. 3B, C). Ratios of dextran penetration versus full capsule thickness were also calculated, which showed increased fractional penetration of dextran in the *lhc1*Δ versus wt or complemented strains. (wt: 0.61±0.03; *lhc1*Δ: 0.72±0.03; *lhc1*Δ+*LHC1*: 0.46±0.03; p<0.05 for *lhc1*Δ versus either wt or complemented strain; Fig. S4 in Text S1). These data show that *LHC1* plays a role in reducing capsule permeability of *C. neoformans* to large molecules.

### Capsular PS of *lhc1*Δ mutant strains of *C. neoformans* exhibits higher dimensions and higher negative charge

Since altered cross-linking or branching of the capsular polymer may affect the dimensions of the *C. neoformans* capsular PS, hydrodynamic sizes of DMSO-extracted capsular PS were determined by dynamic light scattering as described [22]. These data (Fig. 3D) demonstrated two sets of particle distributions as described previously for the DMSO-extracted polysaccharide [23]. Interestingly, the particle distributions from the *lhc1*Δ mutant strain were much larger and more heterogeneous than those from the wt strain, suggesting a role for *LHC1* in reducing capsular PS dimensions.

Zeta potential (ζ) of polysaccharide samples were also determined for the capsular material from the mutant strain. Zeta potential is a measurement of charge and is defined as the electric potential gradient between a boundary liquid in contact with a solid and the mobile diffuse layer in the body of the liquid. Colloidal suspensions having ζ that deviate from zero (>×30 mVolts) have greater solvent hydration and tend to remain in stable suspension, whereas those with values closer to zero tend to aggregate [24]. Using this approach ζ was found to be −28.28±0.30 mV for the wt strain and −34.46±0.61 mV for the *lhc1*Δ mutant (Fig. 3E; p<0.001). This suggests that the larger particles of the *lhc1*Δ mutant seen in the polydispersity profile (Fig. 3E) were also more highly hydrated either by increased cross-links/branching and/or by differences in glucuronic acid availability, the latter suggested by the neutral sugar analysis.

### Deletion of *LHC1* is associated with alterations in higher order polysaccharide structure

We next utilized light scattering to assess higher order capsular structure. These biophysical methods recently demonstrated evidence for branching/cross-linking within the polysaccharide matrix that is difficult to assess by chemical methods alone [10]. For these studies, DMSO-solubilized capsule was analyzed without size fractionation to reduce bias that could be introduced by excluding important capsular constituents. As shown in Table 1, deletion of *LHC1* was associated with changes in a number of macromolecular parameters including average molecular mass (M_w_), radius of gyration (R_g_), hydrodynamic radius (R_h_), mass density and the 2^nd^ virial coefficient (A_2_). Interestingly, while shape factor and A_2_ were restored by complementation, the other parameters were not, suggesting a sensitivity to gene dosing of some of these parameters relative to wt cells by the heterologous insertion of the *LHC1* gene as previously described [25] and examined more recently [26]. The significant increase in M_w_ and R_h_ are consistent with the capsular dimension results (Fig. 3E), demonstrating that the increase in capsule size observed in the mutants is due to the presence of larger polysaccharide molecules. Interestingly, the ratio of R_g_/R_h_, referred to as the shape factor, ρ, was much lower in the mutant than wt and the complemented strain. This was a key parameter demonstrating higher structural complexity of the cryptococcal polysaccharide with low values suggesting higher levels of branching [10]. In addition, the A_2_ coefficient was altered in the mutant. The A_2_ coefficient is a property which describes the interaction strength between the molecule and a solvent, giving insights into the tendency of polysaccharide-polysaccharide interactions in that solvent [27]. Solubilized material from the mutant strain manifested a negative A_2_ value (−2.6±1.1×10^−4^ cm^3^ mol/g^2^) compared to a positive value of both the wt (1.82±0.6×10^−4^ cm^3^ mol/g^2^) and the complemented strain (1.74±0.5×10^−4^ cm^3^ mol/g^2^), suggesting that the strength of molecular-solvent interactions in the mutant strain is lower than the molecular-molecular interactions, relative to the wt strain, and that intra-branch interactions in the mutant are stronger than in the wt strain. Thus, the *lhc1*Δ strain appeared from the biophysical data to produce a population of higher molecular weight capsular PS that showed more solvent hydration and exhibited a greater degree of branching/cross-linkage. To obtain additional structural data, wt and *lhc1*Δ cells were induced for capsule on 1∶10 SAB media and subjected to cryo EM. As shown in Fig. 3F, representative micrographs demonstrated a condensed PS structure in the wt with reduced radius and a larger, more highly branched PS structure in the mutant strain, consistent with the biophysical data. This again suggests a model whereby the capsular adherent lactonohydrolase either directly or indirectly results in the remodeling of secreted polysaccharide particles to reduce particle size and branching/cross-linkages.

**Table 1:** Molecular parameters of capsular PS samples performed by static and dynamic light scattering analysis.

Parameter^1^	Wt	*lhc1*Δ	*lhc1*Δ+*LHC1*
*dn/dc* (620 nm)	0.15	0.15	0.16
*M_w_* (10^7^) (g mol^−1^)	7.9±0.2	12.6±0.3	13.5±0.5
*R_g_* (nm)	147±4	161±3	163±4
*R_h_* (nm)	790±16	1270±20	810±20
Polydispersity	0.43±0.01	0.43±0.01	0.420±0.01
Shape factor	0.19	0.13	0.20
Mass Density (10^5^) (g mol^−1^ nm^−1^)	5.4	7.8	8.3
A^2^(10^−4^) cm^3^ mol/g^2^	1.8±0.6	−2.6±1.1	1.7±0.5

1 The refractive index as a function of concentration (dn/dc) in units of mL/g, average-molecular mass (M_w_), radius of gyration (R_g_), hydrodynamic radius (R_h_), polydispersity, shape factor (R_g_/R_h_), mass density (M_w_/R_g_), and 2^nd^ virial coefficient (A_2_) of capsular PS samples. M_w_, R_g_ data are represented as mean +/− SD of 2 measurements. R_h_ and polydispersity data are represented as mean +/− SE of 10 measurements.

### *LHC1*-dependent changes in antibody binding and phagocytosis by the macrophage-like cell line, J774.16

**Figure 4:**
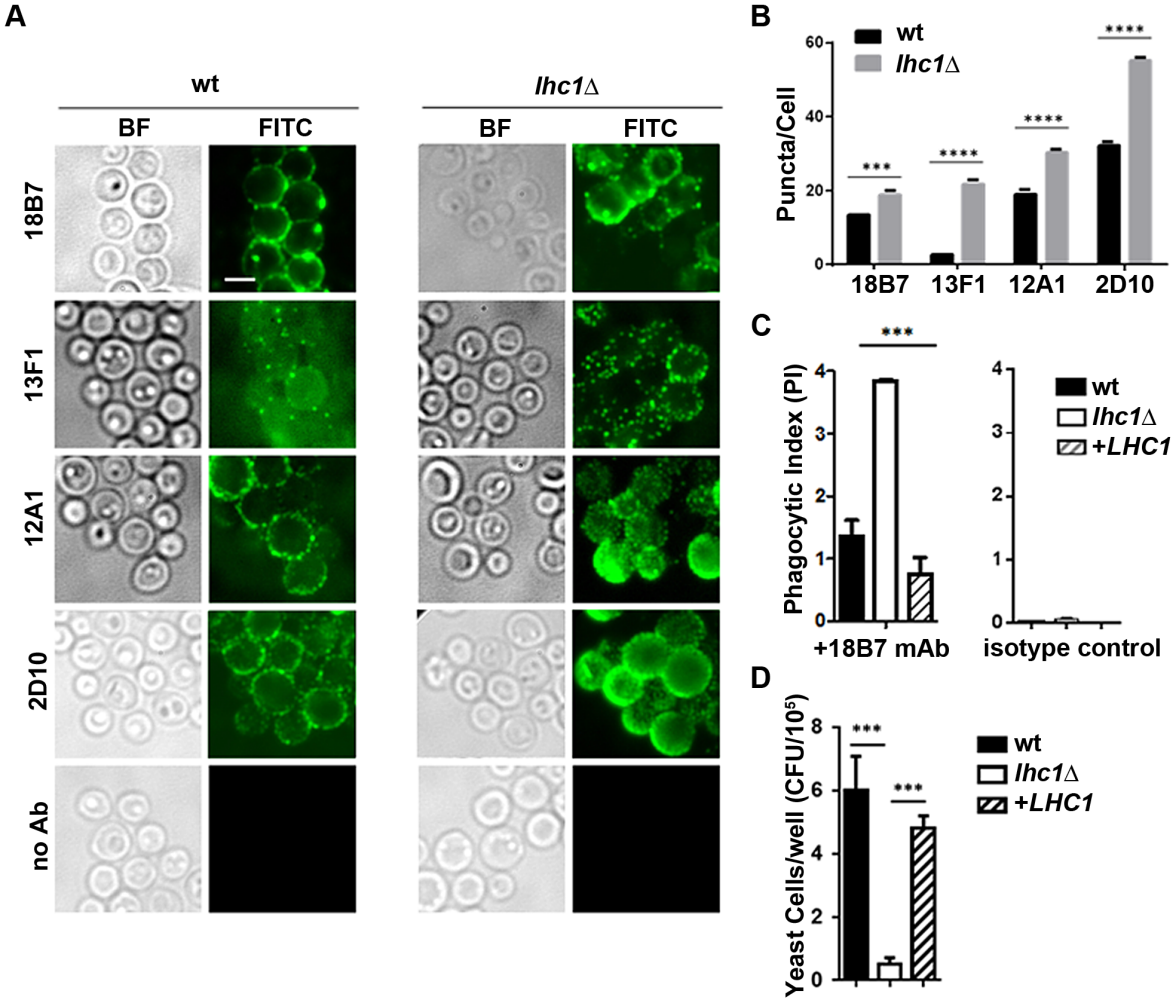
wt and *lhc1*Δ mutant strains of *C. neoformans* differ in antibody binding and antibody mediated phagocytosis by a J774.16 macrophage-like cell line. (A) Indicated strains were prepared and stained with indicated monoclonal antibodies as described in Material and Methods. (B) Puncta from cells labeled as in A were quantified from 50 cells. (C) Indicated strains were opsonized with the indicated antibody and then incubated with J774.16 cell monolayers and phagocytic index determined as in Materials and Methods (N = 4). (D) Fungal Killing Assay: Cells treated as in C, except that incubation was continued for 81 hours and fungal burden assayed by CFU after macrophage lysis (N = 4). *** p<0.001.

To determine the functional significance of the *LHC1*-dependent altered higher order structure in the capsule, we compared binding of mAb to the capsule. Antibodies showed a punctate pattern of binding with subtle but significant differences in the number of puncta observed between the *LHC1* strains (Fig. 4A, B). Antibody negative (Fig. 4A) or isotype controls (data not shown) showed no significant binding. Differences in puncta observed after antibody deposition on *C. neoformans* capsule have been associated with differences in antibody-mediated protection but the mechanism has not been elucidated [28]. Opsonization with the mAb 18B7 yielded increased phagocytosis of the *lhc1*Δ strain by the J774.16 macrophage-like cell line with an almost doubling of the phagocytic index defined as number of fungal cells/macrophage (wt: 1.4±0.3; *lhc1*Δ: 3.85±0.02; *lhc1*Δ+*LHC1*: 0.8±0.3; p<0.02– *lhc1*Δ versus wt or complemented strain; Fig. 4C, left panel), with little phagocytosis evident, using a mouse IgG1 isotype control (Fig. 4C, right panel). Successful phagocytosis by macrophages plays a major role in killing of this facultative intracellular pathogen [29]. Opsonization was unsuccessful with mAbs 12A1 and 2D10 as IgM antibodies are not opsonizing, although these latter two IgM antibodies are capable of opsonizing serotype D *C. neoformans* strains through facilitation of a unusual conformational change in the capsule [30]. Increased antibody opsonization was also associated with decreased survival of the mutant strain after prolonged incubation in J774.16 cells (Fig. 4D). In summary, differences in capsular PS higher order structure as determined by biophysical methods translated into demonstrable changes in antibody-mediated rates of phagocytosis.

### Complement-mediated phagocytosis and virulence is altered in the *lhc1*Δ mutant strain

**Figure 5:**
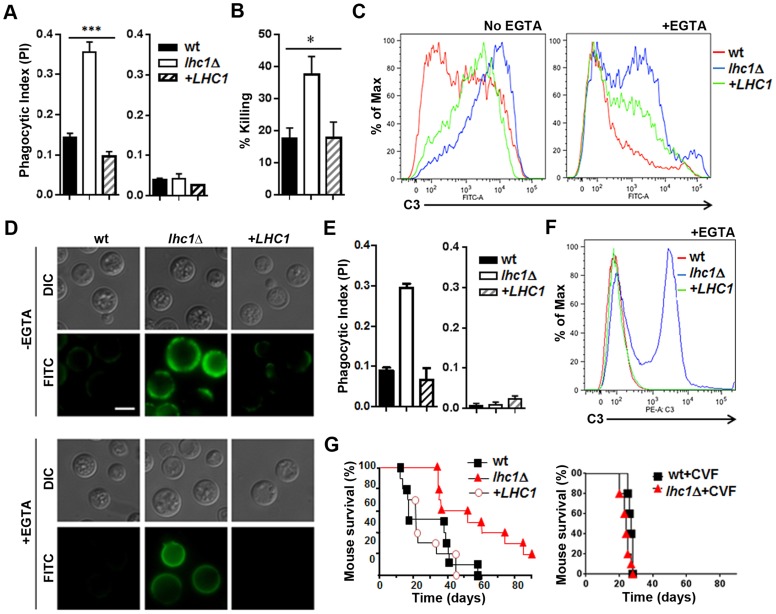
Deletion of *LHC1* results in increased C3 binding from human or mouse serum and reduced virulence in a mouse model. **Human studies, A–D**: (A) Indicated fungal strains were incubated with human serum (left panel) or heat killed serum (right panel) and subjected to phagocytosis using a J774.16 cell line according to Materials and Methods. (B) Strains indicated in B were incubated in the presence of serum and monocytes from a healthy volunteer and percent killing over 4 h measured according to Materials and Methods. (C, D) Indicated strains were incubated in the presence of human serum in the presence (+EGTA) or absence (−EGTA) of EGTA and analyzed by flow cytometry (C) or visualized by fluorescence microscopy (D) using an anti-human C3 antibody. For microscopic evaluation of C3 deposition, FITC-labeled goat anti-human C3 was added and samples resuspended in mounting medium, placed on glass slides and examined under oil immersion at 1000× for C3 deposition (FITC) or using differential interference contrast (DIC). **Mouse studies, E–G:** (E) Indicated strains were incubated with mouse serum (left panel) or heat killed serum (right panel) and subjected to phagocytosis using a J774.16 cell line according to Materials and Methods. (F) Indicated strains were incubated in the presence of mouse serum in the presence (+EGTA) or absence (−EGTA) of EGTA and analyzed by flow cytometry using an anti-mouse C3 antibody. (G) Cumulative mortality in CBA/J mice inoculated intravenously with 1×10^4^ cells of the indicated strains (F-left panel). *p*<0.01 for the comparison of the *lhc1* **Δ** mutant versus either wt or the *LHC1* complemented strain (F-right panel). Cumulative mortality of mice inoculated the same as in F-left panel, except mice were treated with cobra venom factor to deplete serum complement according to Materials and Methods (p = NS).

Similar to that found after antibody opsonization, the *lhc1*Δ mutant strain opsonized with human serum was more readily ingested than the wt strain (Fig. 5A-left panel), an effect that was abolished after heat inactivation (Fig. 5A-right panel), implying complement-dependent opsonization. The capsule serves as a site for deposition of C3 fragments of the alternative pathway of the complement cascade, which promotes *C. neoformans* phagocytosis [31], while the polysaccharide blocks activation of the classical pathway that can occur at the cell wall of avirulent non-encapsulated strains [32]. Incubation of fungal cells with human PBMC's after opsonization with fresh human serum resulted in increased fungal killing (Fig. 5B). Quantitation by flow cytometry demonstrated that C3 deposition was increased in the mutant strain, even after the addition of EGTA to inhibit the classical pathway, but was abolished (data not shown) after heat treatment (Fig. 5C). Complementation with the *LHC1* locus led to a partial yet significant reduction in complement binding. Fluorescence microscopy using antibody to human C3 further demonstrated C3 binding within the enlarged capsule of the *lhc1*Δ strain (Fig. 5D) that was reduced after *LHC1* complementation. Complement binding was heterogeneously deposited on the cells, most likely due to the presence of focal initiation sites, as previously described [32], [33]. Most of the C3 was identified in the form of iC3b (Fig. S6 in Text S1), as previously described [34]. To evaluate the consequences of the reduced *LHC1*-dependent complement binding in *C. neoformans*, we modeled the studies in mice, a species that would also allow testing for virulence [35]. As shown in Fig. 5E, opsonization with mouse serum reproduced the results using human serum and flow cytometry using antibody to C3 again demonstrated increased C3 binding. Inoculation of mice using an intravenous model showed reduced virulence of the *lhc1*Δ strain that was restored after complementation with the wt gene (Fig. 5G-left panel). Interestingly, after depletion of complement using cobra venom factor [33], the differences in virulence between wt and mutant strains disappeared (Fig. 5G-right panel), with a small increase in overall virulence of the wt strain, as previously described after complement depletion [36]. These data suggest a role for *LHC1* in reducing mouse as well as human complement binding to *C. neoformans* and support a role for complement in mediating *LHC1*-dependent mammalian virulence.

## Discussion

A number of formidable pathogenic microbes express PS capsules that are potent virulence factors. Despite their importance in pathogenesis, many aspects of capsular architecture remain poorly understood. The cryptococcal capsule is particularly large and complex, resulting in cells with diameters up to 50 µm in diameter that cannot be ingested by phagocytic cells [37]. The primary structure of the cryptococcal capsule has been well characterized [7]. Xylose and glucuronic acid substituents are attached to a mannose backbone that are readily detectable by NMR spectroscopy. However, given that GXM polymers have masses in excess of 1 mDa, current methods of analytical chemistry cannot reliably detect the rare sugar modifications that may be responsible for tertiary structural complexity [38]. Thus, to identify proteins possibly involved in the modification of the capsular structure, DMSO extraction of intact cells was followed by extensive dialysis and capture on a charged agarose matrix which identified a lactonohydrolase that co-localized with the capsule. Further studies utilizing an mCherry-tagged recombinant protein, biochemical as well immunolocalization confirmed this capsular localization. While the putative Lhc1 sequence does not contain a putative N-terminal leader sequence, several unconventional protein secretion mechanisms have been described including secretion of protein-filled exosomes [39]. Lactonohydrolases are hydrolytic enzymes that cleaves the lactone group within carbohydrates to produce the corresponding organic acid and are expressed by a wide variety of plants and plant-associated fungi but have not been implicated in capsule modifications [40].

Deletion of the *LHC1* locus resulted in a larger capsule that blocked penetration by larger particles comprising India Ink, but allowed increased diffusion of a fluorescent dextran polymer. This phenotype was associated with a reduction in virulence in a mouse model and the increased capsule size persisted during infection in brain. Classical analytical approaches [8] yielded only subtle differences between the wt and mutant strain, including a slight increase in backbone sugars such as mannose and xylose [38] as well as branching glucuronic acids by neutral sugar analysis. NMR spectroscopy also suggested only subtle differences that reflected retention of much of the mannose backbone and primary branching, with a similar mannosyl∶xylosyl∶glucuronic acid ratio, but potentially stronger cross-linkage between GXM chains, given the resistance of the mutant PS to sonication. These results differed somewhat from the elemental analysis and may be due to the preparation of the material for NMR spectroscopy which used soluble GXM, rather than DMSO-extractable polysaccharide material used for the elemental analysis.

Such subtle changes in primary structure as well as increased diffusion of dextran dye in the mutant suggested a role for *LHC1* in capsular tertiary structure that could result in a larger, more open structure in the mutant strain. Biophysical methods were thus used to study DMSO-extractable PS of the cryptococcal capsule that comprise the outer interface with phagocytes during infection [21] and also contained the adherent Lhc1 protein. Notable was the larger molecular mass and increased particle size of the PS from the *lhc1*Δ mutant by polydispersity measurements, which correlated with the overall larger capsule size visualized by India ink microscopy. Previous work had noted an association between extracted particle size and overall capsule size between cryptococcal strains [23]. In addition, the larger negative zeta potential of PS from the mutant strain suggested a more solvated surface for these larger molecules and a tendency towards less aggregation that could provide a more open structure for antibody and complement deposition. The mutant strain PS was also found to have a lower shape factor ρthan wt, determined by the ratio of R_g_, the radius of gyration and R_h_, the hydrodynamic radius, which provides an important measure of whether a molecule is loosely linear or branched [41], [42]. *C. neoformans* wt strains tend to produce PS which have low ρ, more similar to branched polysaccharides such as glycogen and amylopectin [10]. This suggests that the surface structure of the larger *lhc1*Δ mutant PS contains an even more highly-branched/cross-linked surface component that is also more efficiently solvated. Small changes in the number of glucuronic acid residues in the mutant strain, detected in the composition assay, could have contributed to this increased surface hydration, either alone or in combination with a more highly branched surface structure. A lower ρ value could also reflect aggregation of components of the PS capsule [43], but would be inconsistent with the more highly negative zeta potential value that suggests a lower tendency towards aggregation. In addition, examining the two values contributing to ρ, most of the change was due to that of the radius of hydration, which is a measure of the hydrated zone surrounding a given polysaccharide particle, with very little change in the radius of gyration, again suggesting a more branched, hydrated structure.

**Figure 6:**
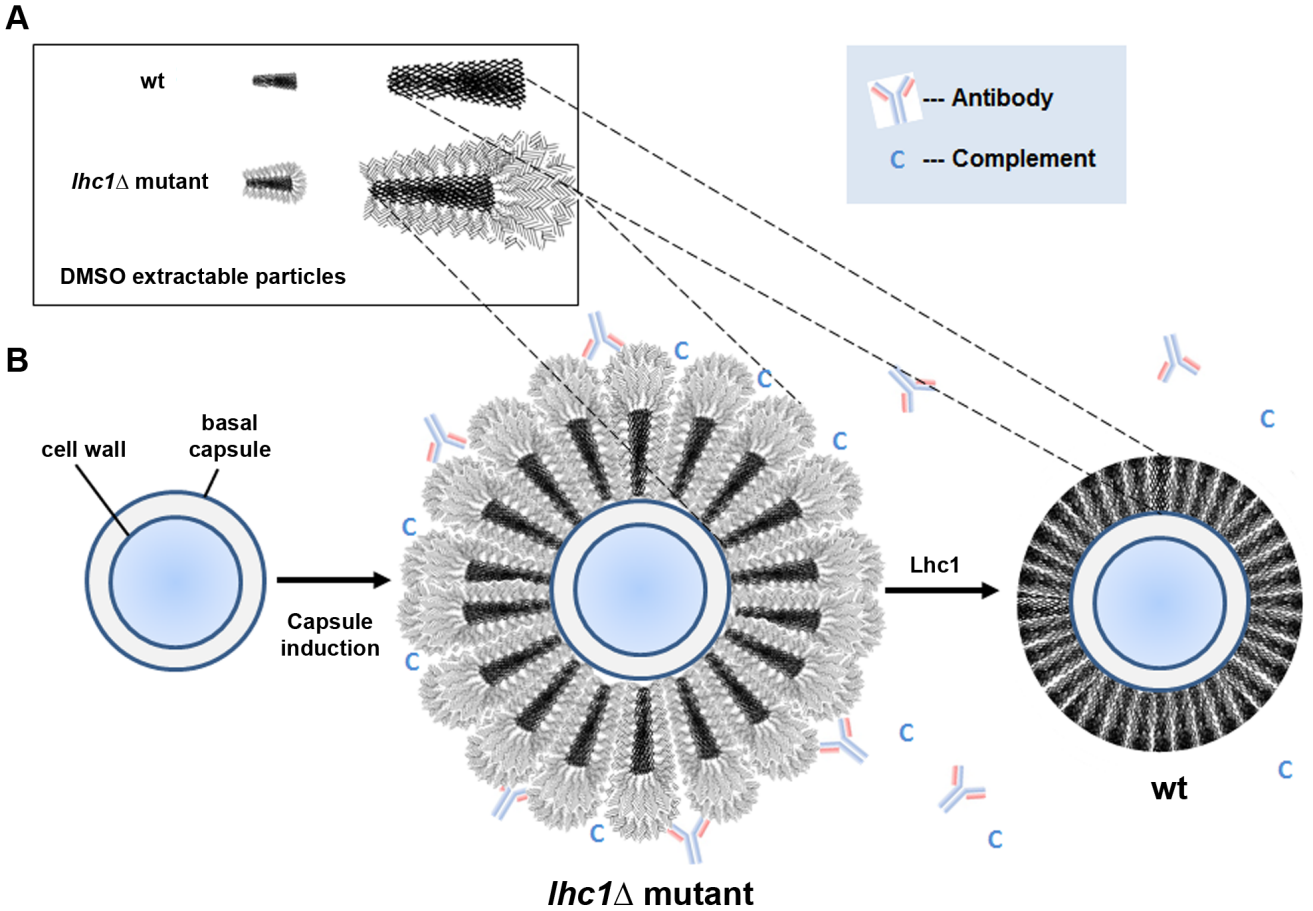
Scheme of working model of capsule modification by Lhc1. (A) Data from particle analysis of DMSO-solubilized PS and cryo-electron microscopy suggests hydrolytic, Lhc1-dependent processing of two populations of particles during capsule induction. Hydrolysis of outer branching units within the PS surface structure reduces both the size and overall branching as the wt particle adopts a surface having reduced hydration, radius of hydration and increased zeta potential. (B) Model of outer capsular synthesis. In the absence of Lhc1, unprocessed PS units with increased branching and increased surface hydration provide sites for antibody and complement binding, resulting in increased phagocytosis. Action of Lhc1 serves to provide a less hydrated, more compact capsule, resulting in better exclusion of innate immune products, reducing phagocytosis and increasing virulence potential. Events are not intended to be sequential; PS synthesis and *LHC1*-dependent remodeling likely are simultaneous. Representation does not intend to imply that PS particles are symmetrical or have regularly-spaced branches.

Combining the results of this study with prior contributions from several laboratories and more recent studies of the capsular architecture suggest a tentative model that may help to illustrate the modifications of *C. neoformans* capsular PS by Lhc1 and exclusion of anti-microbial products (Fig. 6). The location of a hydrolytic lactonohydrolase within the PS capsular structure and the smaller size of the PS particles in the wt strain suggest that the enzyme either directly or indirectly plays a role in remodeling secreted PS fibrils (Fig. 6A). Recent data has suggested that the capsule is assembled by non-covalent binding of PS fibrils that have a branched structure [10]. Hydrolysis of outer branching units within PS surface structure by Lhc1 in wt cells (Fig. 6B, right panel) could reduce the size of the capsular PS (compared to that of the *lhc1*Δ mutant; Fig. 6B, middle panel), and its overall branching if the outer hydrated segments were also highly branched. This would result in a reduced hydrated surface with a smaller radius of hydration and a less negative zeta potential in the wt cells, resulting in a smaller, more compact capsule. This structure was supported by cryo-scanning electron microscopy which demonstrated a compact structure in the wt cells composed of truncated fibrils, whereas the mutant strain demonstrated a much more open lattice composed of larger fibrils, likely representing the larger PS demonstrated by the polydispersity measurements. The poor fitting and better solvation of the unprocessed mutant PS within the capsular gel lattice in the *lhc1*Δ mutant thus resulted in increased diffusion of dextran particles and greater penetration by anti-microbial products or exposure of binding epitopes. PS processing by Lhc1 could be due to the presence of trace lactone units forming some of the PS crosslinks or other carbohydrate linkages specifically tailored to the hydrolytic activity of Lhc1. However, identifying trace lactone groups within a large polysaccharide background is particularly difficult. To put this problem in perspective, the molecular mass of GXM is >1 mDa [44]; thus, detection of rare lactone groups is probably beyond the current analytical horizon. If true, the occurrence of lactone-dependent crosslinking would imply that this step of capsular assembly occurs in the extracellular space and that random concentration gradients of reagents involved in such processes within the capsule could contribute to the remarkable antigenic variability reported within the capsule structure [45]. Localization of the protein to the capsule, suggested by the method of isolation, western blots of cell fractions and immune- and fluorescence-microscopy, also supports a remodeling role in capsular synthesis. Indeed, the mCherry-Lhc1 localization studies appear to suggest production of the enzyme during capsule induction at the outer region of the capsule which is the region that is thought to represent an inducible outer layer that forms on top of an older inner capsule layer [46]. Lhc1 protein production during capsular induction also suggests such an extracellular mechanism. However, an indirect or regulatory role cannot be ruled out. Interestingly, the gene has previously been found to be induced in response to hypoxia, which may facilitate virulence in the relatively hypoxic environment of infected tissue [47].

Using either mAb 18B7 or serum as opsonin, the *lhc1*Δ mutant exhibited higher rates of phagocytosis than the wt or complemented strain. Patterns of antibody binding by the IgM mAb 12A1 and 2D10 were altered in the *lhc1*Δ mutant, whereas only subtle differences were noted using the IgG mAb, 18B7, which may have been due to the smaller size of the IgG antibody or to differences in epitopes to which they bind. As suggested by the serum-dependent phagocytosis data, more complement was deposited on the capsule of the *lhc1*Δ mutant than the wt strain both in the presence or absence of EGTA, which blocks the classical pathway. The increased deposition of C3 on the *lhc1*Δ capsule was accompanied by reduced virulence in a mouse model which was reversed by depletion of complement by cobra venom factor in the mice, suggesting a role for complement in the differential virulence between mutant and wt strains in complement-sufficient mice. There is strong evidence supporting a role for the alternative pathway in innate protection against *Cryptococcus*, since animals deficient in C3 but not C4 have reduced survival [48], [49]. Deposition of complement is important for efficient opsonization of the fungus, rather than direct killing by production of a membrane attack complex [50]. Complement fragments iC3b were also increased on the *lhc1*Δ mutant, which are important opsonic ligands and forms rapidly on the cryptococcal capsule after complement deposition [34]. Reducing alternative pathway activation by *LHC1* thus facilitates a role for *C. neoformans* as an effective pathogen. In summary, the current studies demonstrate a role for *LHC1* in the remodeling of the polysaccharide capsule of *C. neoformans* that represents a unique mechanism of virulence optimization among pathogenic microbes.

## Materials and Methods

### Ethics statement

Research involving human participants was approved by the NIAID intramural institutional review board and written, informed consent was obtained from all study participants before participation and was conducted according to the principals in the Declaration of Helsinki. All experimental procedures involving animals were conducted under guidelines of the National Institutes of Health and protocols approved by the Institutional Animal Care Committees (IACUC) of the Intramural NIH/NIAID and the University of Illinois at Chicago.

### Fungal strains, plasmids and media

*Cryptococcus neoformans* ATCC 208821 (H99) was a generous gift of J. Perfect. Strain H99 *ura5*, [51] was employed as a recipient strain for deletion and expression studies and were maintained on media described in Supplemental Material and Methods. Plasmid pCIP containing the *URA5* gene was a kind gift of K.J. Kwon-Chung.

### Isolation of capsular proteins from DMSO-extracted capsule

*C. neoformans* strain B-3501 was grown at 30°C in RPMI supplemented with 2% glucose to stationary phase. Fifty grams of cells were harvested, washed extensively in 10 mM sodium phosphate, pH 7.0 and exchanged into dimethylsulfoxide as described [11] and incubated overnight with shaking at 37°C. Supernatant was harvested and dialyzed extensively against 10 mM sodium phosphate buffer, pH 7.0, followed by recovery of protein by passage of the dialysate on a 1 ml (DEAE)-Sepharose column. The column was extensively washed in 10 mM sodium phosphate, pH 7.0 and eluted with the same buffer containing 0.5 M NaCl. Eluate was again dialyzed and subjected to PAGE. Proteins were then transferred to nitrocellulose membranes and subjected to automated protein sequencing after protease Lys-C digestion as described [52].

### Disruption and complementation of *LHC1* in *C. neoformans*

Standard methods were used for disruption and complementation of the *LHC1* gene in strain H99 as described previously using two PCR-amplified fragments and a 1.3-kb PCR fragment of the *URA5* gene previously described to effect a 1.4-kb deletion within the *LHC1* coding region (see Supplemental Materials and Methods in Text S1) and was complemented using a 3.6-kb genomic fragment of the *LHC1* gene.

### Analysis of lactonohydrolase activity

Fungal cells or recombinant enzyme were assayed for hydrolysis of the aliphatic lactone D-pantonylactone using a previously-described method [17] after induction in minimal media (0.1% glucose, 1 g/L asparagine, 20 mM sodium phosphate, 1 g/L YNB without amino acids and ammonium sulfate) for 3 days 30°C (see Supplemental Materials and Methods in Text S1) and assayed for hydrolysis of 100 mM D-pantonylactone by high performance liquid chromatography by reference to standard D-pantoic acid.

### Preparation of a recombinant lactonohydrolase and generation of antibody to Lhc1

Full-length and an N-terminal fragment of lactonohydrolase was expressed in *E. coli* as a fusion protein with maltose-binding protein by using the pIH902 expression system (New England Biolabs, Beverly, Mass.) and the recombinant maltose-binding protein–lactonohydrolase fusion protein (MBP-Lhc1) purified on amylose-Sepharose according to the manufacturer's directions as described in Supplemental Materials and Methods. Mice were immunized by a standardized protocol with either MBP-Lhc1 or MBP alone as control and MBP antibodies removed as described in Supplemental Materials and Methods.

### Immunolocalization studies

Histopathological material was prepared, embedded and fixed and incubated with either anti-Lhc1 or anti-MBP antibody and observed using fluorescence microscopy as described in Supplemental Materials and Methods.

### Measurement of capsular size and permeability

To induce capsule, yeast cells were grown in 3 mL of RPMI in a 12-well plate incubated in a CO_2_ enriched environment (GasPak EZ CO_2_, Becton Dickinson) in a 37°C water jacketed incubator for 4 days. Alternatively, capsule was induced by growth on 1∶10 dilutions of Sabaraud's media at 30°C or RPMI agar for the indicated times and Lhc1 transcript was measured by quantitative RT-PCR and is described in Supplemental Material and Methods. TMR-Dextran 2,000 kDa (TMRD, Invitrogen) staining of *C. neoformans* capsule on cells grown under capsule-inducing conditions was performed as described previously [21]. The distance between the outside of the cell wall and the staining front was measured using Slidebook software. One-way ANOVA was used to assess statistical significance among the strains; Tukey's t test was used to perform pairwise analyses post-hoc.

### Glycosyl composition of polysaccharide

Glycosyl composition analysis was performed on DMSO-extracted capsule by combined gas chromatography/mass spectrometry (GC/MS) of the per-*O*-trimethylsilyl (TMS) derivatives of the monosaccharide methyl glycosides produced from the sample by acidic methanolysis as described [53].

### Analysis of capsule by nuclear magnetic resonance spectroscopy

Soluble glucoroxylomannan (GXM) was prepared and analyzed by NMR as previously reported [54]. Briefly, Isolated soluble GXMs were sonicated and lyophilized three times before being dissolved in 0.5 ml of 99.96% D_2_O (Sigma, St. Louis, Mo.) for nuclear magnetic resonance (NMR) analysis by standard methods [55]. NMR spectra were acquired with a Bruker Avance 600 NMR spectrometer, using a 5-mm (1H, 13C) inverse-detection dual-frequency probe, operating at 600.13 and 150.913 MHz, respectively as described.

### Biophysical analysis and crypto-electron microscopy of DMSO-extracted capsular polysaccharide

Capsular PS samples from H99 wt, *lhc1Δ and lhc1Δ LHC1* strains were isolated by DMSO extraction, prepared and subjected to light scattering analysis as described [10] and is described further in Supplemental Materials and Methods. Cryo-electron microscopy was performed on indicated cells and is described further in Supplemental Materials and Methods.

### Virulence factor expression and virulence studies

Capsule was measured by microscopy after the fungal cells were suspended in India ink [56], urease production by incubation on Christensen's agar [57], and laccase by melanin production on nor-epinephrine agar [58]. Virulence studies were conducted according to a previously described intravenous mouse meningoencephalitis model [59] using 10 CBA/J mice for each *C. neoformans* strain. In a second experiment, animals were treated with cobra venom factor (CVF) according to Shapiro et al. [36], described in Supplemental Materials and Methods.

### Phagocytosis and killing assays

Phagocytosis and fungal killing assays were conducted using J774.16 cells by the method of Shapiro et al [36] or the method of Miller and Mitchel [60] using human PBMCs and is described in Supplementary Materials and Methods. The phagocytosis index was determined by microscopic examination of the number of fungal cells ingested or adherent divided by the number of total macrophages.

### Detection of C3 binding by flow cytometry and fluorescence microscopy

Fungal binding of C3 from mouse and human serum was determined by flow cytometry using goat anti-mouse C3-FITC (ICN), anti-human C3-FITC (Invitrogen) or anti-human iC3b (Quidel) in the presence or absence of EGTA by the method of [33] using a Becton Dickenson LSR Fortessa flow cytometer. C3 binding was determined by fluorescence microscopy using a FITC-labeled anti human C3 (Invitrogen) and visualized by fluorescence microscopy.

### Statistics

The capsule radius was measured in India ink experiments using 10 cells for each strain and means compared using ANOVA with Tukey's test post hoc. Errors were expressed as standard error of the mean (SEM). Statistical significance of mouse survival times was assessed by Kruskall-Wallis analysis (ANOVA on Ranks). Statistical analyses for capsular biophysical measurements were carried out using Bi-ZPMwA Zimm Plot Software (Brookhaven Instruments).

90Plus/BI-MAS Software was used for effective diameter and polydispersity parameters (Brookhaven Instruments). Comparison of phagocytic index was performed by a non-parametric t-test with Welch's correction. Plots, curve fits, Pearson or Spearman correlations (*r*), and statistical analysis were performed using GraphPad Prism version 5.0a, GraphPad Software, San Diego, California, USA.

## Supporting Information

Text S1 Supporting information file containing Table S1 showing sequences of DMSO-solubilized proteins, Table S2 showing glycosyl composition analysis (mole %), Figure S1 showing capsule diameter is dependent on *LHC1*, Figure S2 showing induction of Lhc1-mCherry in ASN and RPMI media, Figure S3 showing quantitative RT-PCR of *LHC1* under indicated conditions, Figure S4 showing aggregate TMRD diffusion measurements, Figure S5 showing western blot of cryptococcal extract using anti-Lhc1 antigen-purified antibody, Figure S6 showing deletion of *LHC1* results in increased iC3b binding from human serum. And Supplemental Materials and Methods containing fungal strains, plasmids and media, disruption and complementation of *LHC1* in *C. neoformans*, Lhc1-mCherry fusion protein, quantitative RT-PCR experiments, western blot analysis, analysis of lactonohydrolase activity, preparation of a recombinant fragment and full-length lactonohydrolase and generation of anti-Lhc1 antibody, Immunolocalization studies, biophysical analysis of DMSO-extracted capsular polysaccharide, Cryo-Scanning Electron Microscopy (Cryo-SEM). Immunolocalization studies, phagocytosis assay, fungal killing assay by human monocytes, fungal killing by J774A.1 cells, mouse virulence model with the addition of cobra venom factor, detection of C3 and iC3b binding by flow cytometry, and Supplemental References.
(DOCX)
