# Supplementary material for: A Role for LHC1 in Higher Order Structure and Complement Binding of the Cryptococcus neoformans Capsule
Source: PLoS Pathog. 2014 May 1;10(5):e1004037. doi: 10.1371/journal.ppat.1004037 (PMC4006888; doi:10.1371/journal.ppat.1004037)
Supplement: Text S1 — Supporting information file containing Table S1 showing sequences of DMSO-solubilized proteins, Table S2 showing glycosyl composition analysis (mole %), Figure S1 showing capsule diameter is dependent on LHC1, Figure S2 showing induction of Lhc1-mCherry in ASN and RPMI media, Figure S3 showing quantitative RT-PCR of LHC1 under indicated conditions, Figure S4 showing aggregate TMRD diffusion measurements, Figure S5 showing western blot of cryptococcal extract using anti-Lhc1 antigen-purified antibody, Figure S6 showing deletion of LHC1 results in increased iC3b binding from human serum. And Supplemental Materials and Methods containing fungal strains, plasmids and media, disruption and complementation of LHC1 in C. neoformans, Lhc1-mCherry fusion protein, quantitative RT-PCR experiments, western blot analysis, analysis of lactonohydrolase activity, preparation of a recombinant fragment and full-length lactonohydrolase and generation of anti-Lhc1 antibody, Immunolocalization studies, biophysical analysis of DMSO-extracted capsular polysaccharide, Cryo-Scanning Electron Microscopy (Cryo-SEM). Immunolocalization studies, phagocytosis assay, fungal killing assay by human monocytes, fungal killing by J774A.1 cells, mouse virulence model with the addition of cobra venom factor, detection of C3 and iC3b binding by flow cytometry, and Supplemental References. (DOCX) [file ppat.1004037.s001.docx]

**Supplemental Tables**

**Table S1: Sequences of DMSO-solubilized proteins**

| H99 database | est MW | Sequence | BLAST |
| --- | --- | --- | --- |
| CNAG_01562 | 32 kDa | AKPTTYDEGYLERYDA | conserved hypothetical |
| CNAG_00920 | 68 kDa | IYVTGESYAGRYVPYIGAAMLDAQDK | Kex1 |
| CNAG_04753 | 52 kDa | DFAVLTTVLPSYEFXGSSLFVPPGT | Lactonohydrolase |

Table S2; Glycosyl Composition analysis (mole %)

| Glycosyl Residue | wt | *lhc1*∆ |
| --- | --- | --- |
| Xylose | 19 | 32 |
| Glucuronic Acid | 1.2 | 1.6 |
| Mannose | 20 | 37 |
| Galactose | 0.8 | 1.7 |
| Glucose | 60 | 28 |

**Supplemental Figures**

**Figure S1: Capsule diameter is dependent on *LHC1*.** Cells were grown under the indicated media for 1 day, followed by Indian Ink microscopy according to Materials and Methods. YPD agar 1 day (YPD), 0.1% glucose, 1 g/L asparagine (ASN) 1:10 SAB agar (SAB), or RPMI agar (RPMI). Lower panel indicates capsular size, N = 20; * p < 0.05.


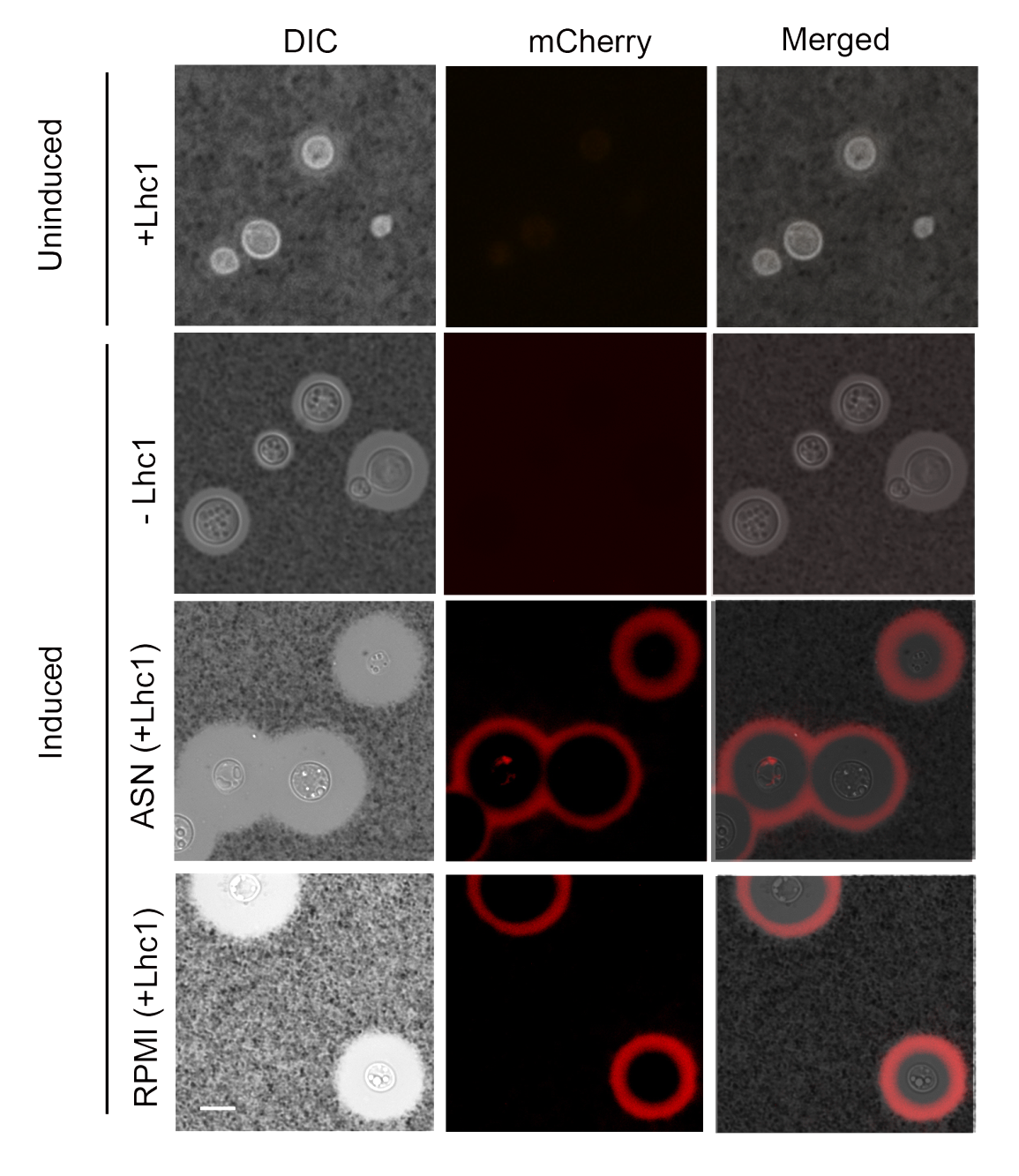


**Figure S2: Induction of Lhc1-mCherry in ASN and RPMI media.** A *C. neoformans lhc1∆* strain complemented with a vector expressing Lhc1-mCherry (+Lhc1) or empty vector alone (-Lhc1) was grown in YPD (uninduced) or induced on the indicated media as described in Materials and Methods.

**Figure S3: Quantitative RT-PCR of *LHC1* under indicated conditions.** Indicated cells were incubated in YPD liquid to mid-log phase (YPD) or induced by incubation for 24 h on the indicated media as in Fig. S1 (N = 3 independent experiments). * p<0.05, ** p<0.01, *** p<0.001

**Figure S4: Aggregate TMRD diffusion measurements** (N = 100, mean +/- SEM).

**
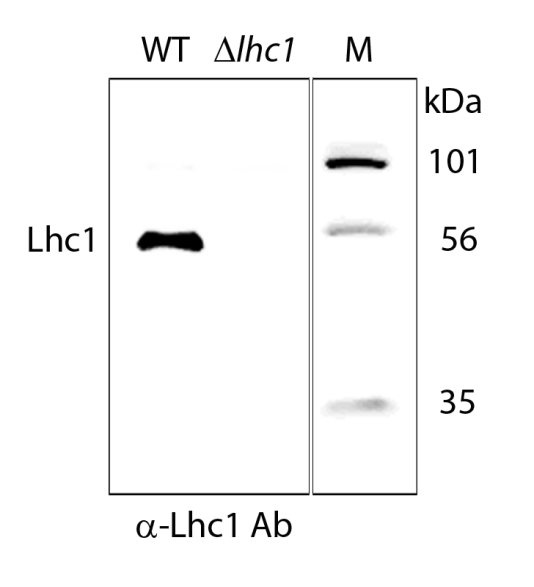
**

**Figure S5: Western blot of cryptococcal extract using anti-Lhc1 antigen-purified antibody.** Fungal cells were induced by growth on 1:10 SAB for 3 days, then subjected to glass bead homogenization and SDS-PAGE and western blot (20 µg/lane total protein) using affinity-purified anti-Lhc1 antibody at 1:300 dilution and secondary HRP-labeled anti mouse (1:1000).

**Figure S6: Deletion of *LHC1* results in increased iC3b binding from human serum.** Indicated fungal cells were incubated in the presence of human serum and analyzed by flow cytometry using an anti-human iC3 antibody as described in Materials and Methods.

**Supplemental Materials and Methods**

**Fungal strains, plasmids and media**

*Cryptococcus neoformans* ATCC 208821 (H99) was a generous gift of J. Perfect. This strain is recovered each time from the original frozen stock each time prior to transformations or virulence studies to minimize genetic drift. Strain H99 *ura5*, [[1](#_ENREF_1)] was employed as a recipient strain for expression studies. Cells were maintained on yeast peptone agar (YPD; 2% glucose, 2% bacto peptone, 1% yeast extract). The asparagine minimum selective media used for transformant selection, capsule induction and for detection of laccase production have been previously described [[2](#_ENREF_2)]. Plasmid pCIP containing the *URA5* gene was a kind gift of K.J. Kwon-Chung.

**Disruption and complementation of *LHC1* in *C. neoformans***

Standard methods were used for disruption and complementation of the *LHC1* gene as described previously [[3](#_ENREF_3)]. Briefly, to make the deletion construct, two PCR-amplified fragments of *C. neoformans* *LHC1*  (using primers: LHC1-396S-Xba, 5’- GCC GCC TCT AGA TAC TCT AGG CAA ACA ATT A and LHC1-1010A-RI, 5’- GCC GGA ATT CAT TAG GAT CAA GCA TGT AAA), and (primers: LHC1-1757S-Bgl, CTT ATC GGA AAG ATC TAC CTC G and LHC1-2199A-Xho, 5’- GCC GCC CTC GAG GGG GAG CCG AAA TA), the first digested with *Xba* I and *Eco R* I and the second digested with *Bgl* II and *Xho* I, was mixed with a 1.3-kb PCR fragment of the *C. neoformans* *URA5* gene described previously [[4](#_ENREF_4)], digested with *Bgl* II and *Eco R* I and ligated to BlueScript SK digested with *Xba* I and *Xho* I. The final disruption allele with a 1.3-kb *URA5* marker flanked on either side by a 500-bp DNA sequence homologous to genomic regions of the *LHC1*  gene was PCR-amplified and introduced into H99FOA cells via a biolistic approach [[5](#_ENREF_5)] to effect a 1.4-kb deletion within the *LHC1*  coding region. Transformants were screened for potential *LHC1* deletion mutant by a PCR approach and the specific disruption of the *LHC1*  gene in candidate mutants was verified by Southern blot analysis. To complement the *lhc1∆*  mutant strain, a 3.6-kb genomic fragment encompassing the full ORF of *LHC1* plus ~1200-bp of the 5’ promoter region was PCR-amplified using a primer set of LHC1-1689S-Mun (5’- GCC GAC CAA TTG GTC GGG GGA ACT CGC) and LHC1+1882A-Mun (5’- GCG GCG CAA TTG GCC TCA GGA TCC CAT CCT GA) and then cloned into a modified Bluescript SK vector (Stratagene) containing the 2-kb hygromycin-B- resistance gene under the control of a cryptococcal actin promoter [[5](#_ENREF_5)] to generate the complementation construct, which was introduced into *lhc1∆* mutant cells by electroporation as previously described [[3](#_ENREF_3)] and transformants were selected on hygromycin-containing YEPD agar plates (150 u/ml). Heterologous genomic insertion of the wt *LHC1* locus and retention of the *URA5* deletion construct in the complement was verified by uncut Southern and PCR.

**Lhc1-mCherry fusion protein**

The cryptococcal shuttle vector pORA-rKNUT [[6](#_ENREF_6)] was used to express a fusion between the Lhc1 protein and a synthetic mCherry protein (Cneo-mCherry), utilizing *C. neoformans* codon usage produced using standard methods [[7](#_ENREF_7)]. The plasmid was digested with *Pst* I, and a PCR-amplified fragment of the H99 *LHC1* gene containing promoter region (obtained using primers LHC1pro-NheI-s; AGG ACG CTA GCC TGG TAT GCT GCT TAC CTA TG and LHC1-NheI-wo ter-a; ATA TAT GCT AGC CGC CTC AGG ATC CCA TCC TG) was digested with *Nhe*I and ligated into compatible sites to produce YP160. The plasmids were recovered, the sequences were verified, and the plasmids were linearized with *Sce*I and transformed into *C. neoformans* H99 Matα *lhc1*Δ cells by electroporation using standard methods [[8](#_ENREF_8)]. Cells incubated with this vector are retained even under negative pressure [[9](#_ENREF_9)]. In addition, all cells preparations grown on non-selective media are assayed at the end of each experiment by simultaneous inoculation of selective and non-selective plates to verify >90% retention of plasmid.

**Quantitative RT-PCR experiments**

*C. neoformans* strains H99 and B3501 were grown on ASN (0.1% glucose, 1 g/L asparagine (ASN) 1:10 SAB agar (SAB), or RPMI agar containing Rosewell Park Memorial Institute media containing 15 g/L agar (RPMI). Lower panel indicates capsular size, SAB, and RPMI media. Real-time PCR was performed using a primer set of LHC1 (LHC1-RT-370s; CGG AAG CTC TCT GTT TGT TC and LHC1-RT-600a; TTA TAC CAA ACA ACA GCT TCG), and *ACT1* (Act1RT-SS-For; GGT ATT GCC GAC CGT ATG CA and Act1RT-SS-Rev; TCC ACA TCT GCT GGA AGG TAG A) as a control. Reverse transcription was performed on DNase-treated RNA using the iScript kit (Bio-Rad Laboratories), according to the manufacturer's protocol. PCRs were set up using iQ SyberGreen SuperMix (Bio-Rad Laboratories), according to the manufacturer's protocol. qRT-PCR was performed using a Bio-Rad iCycler (MyiQ2).

**Western blot analysis**

Cells of the wt and ∆*lhc1* strains were grown to stationary phase in YPD broth. Cells were harvested and boiled with protein loading buffer. The lysates were cleared by centrifugation at 15 000 *g* for 5 min. Total protein was run on a 4–12 % SDS-PAGE gel and transferred to a nitrocellulose membrane. Lhc1 was detected using α-Lhc1 antibody. Antibody reactivity was visualized using Super Signal West Pico reagent, as recommended by the manufacturer (Pierce).

**Analysis of lactonohydrolase activity**

Fungal cells were assayed for hydrolysis of the aliphatic lactone D-pantonylactone using a previously-described method [[10](#_ENREF_10)]. Briefly, fungal cells were grown in minimal media (0.1% glucose, 1 g/L asparagine, 20 mM sodium phosphate, g/L 0.67 YNB without amino acids and ammonium sulfate) for 3 days 30^o^C and concentrated by centrifugation. Alternatively 100 µg of purified full-length Lhc1-MBP (described below) was assayed in 1 ml of the same buffer. Cells or recombinant protein were incubated in the presence of 100 mM D-pantonylactone in 10 mM PIPES buffer, pH 7. 0 and the reaction terminated by centrifugation and addition of methanol in a 1:1 volume, then storage at -80^o^C until analysis. Analysis was performed by high pressure liquid chromatography using a C-18 5m, 4.6 x 250 mm Ultrasphere column using an eluent of 13% methanol (by vol.) in dilute phosphoric acid (pH 2.5) at a flow rate of ml/min, monitoring at 210 nm. Identification of products was made by comparison to retention times of authentic D-pantonyl lactone and pantonoic acid, the latter made by hydrolysis of the lactone by sodium hydroxide hydrolysis at 100^o^C.

**Preparation of a recombinant fragment and full-length lactonohydrolase and generation of anti-Lhc1 antibody**

N-terminal Lhc1 fragment: An N-terminal fragment of lactonohydrolase was expressed in *E. coli* by using the pIH902 expression system (New England Biolabs, Beverly, Mass.). A 740-bp fragment of *LHC1* obtained by PCR with *Pfu* polymerase (Stratagene, La Jolla, Calif.), H99 genomic DNA as a template and primers LHC1+1-XBA: 5’-TAATTCTAGAATGGCTACGAATAAGAATCCCG and

LHC1+740PST: 5’-TAATCTGCAGTTAAGCGCTTTTAGCACCTGCATTC

was endonuclease-digested with *Eco* RI and *Xba* I and inserted into compatible sites of pIH902.

Full length Lhc1: Full length Lhc1 was expressed using the pMAL-c expression after insertion of a full-length 1404-bp fragment of the *LHC1* coding region obtained by PCR with *Pfu* polymerase (Stratagene, La Jolla, Calif.), H99 cDNA as a template and primers Lhc1-ORF-XbaI-s (5-TAA TTC TAG AAT GGC TAC GAA TAA GAA TCC CG-3’) and Lhc1-ORF-PstI-a (5’-TAA TCT GCA GTT AAG CGC TTT TAG CAC CTG CAT TC-3’) was endonuclease-digested with *Xba* I and *Pst* I and inserted into compatible sites of pMAL-c. The recombinant maltose-binding protein–lactonohydrolase fusion proteins (MBP-Lhc1) and control MBP expressed from the empty plasmid were expressed and purified on amylose-Sepharose according to the manufacturer’s directions.

Immunization**:** Male BALB/c mice, 18 to 20 weeks old (National Cancer

Institute, Rockville, Md.), were injected intraperitoneally with 50 mg of either full-length recombinant lactonohydrolase protein (for immunofluorescence in Fig. 2E) or the N-terminal fragment (immunoblot, Fig. 2C) in a 1:1 (vol/vol) emulsion of complete Freund’s adjuvant (Sigma) and phosphate-buffered saline (PBS). At week 4 after immunization, mice were boosted with 25 mg of the respective lactonohydrolase protein in a 1:1 (vol/vol) emulsion of incomplete Freund’s adjuvant (Sigma) and PBS. After the immune response was induced, the mice were bled from the retro-orbital plexus, and their sera were analyzed for antibodies to the recombinant protein by western blot. The mouse with the highest antibody titers was boosted three times with 50 mg in PBS at a 2-wk intervals, and serum recovered for immunolocalization studies. To reduce background immune reactivity for immunofluorescence studies (Fig. 2E) antisera directed against full length Lhc1 was adsorbed on amylose-Sepharose to remove anti-MBP antibodies. Flowthrough was dialysed against PBS for 20 h and tested by western blot which demonstrated a single band against wt *C. neoformans* extract which was not present using equivalent extracts from a *lhc1∆* strain (Fig. S5).

**Immunolocalization studies**

Western blots. *C. neoformans* cells were grown in 1:10 Sabaroud media to stationary phase, washed and homogenized by glass bead homogenation [[11](#_ENREF_11)]. Cell wall/capsule and cytosol fractions were separated by centrifugation at 12,000 g x 10 min and cell wall/capsule proteins were extracted from the pellet by a 10 min extraction with SDS buffer (50 mM Tris HCl, pH 8.0, 0.1 mM EDTA, 2% SDS, 10 mM DTT) and clarified by centrifugation at 12,000 *g* for 5 min. 20 mg aliquots of protein were subjected to SDS/PAGE-western blot using 1:500 of the primary serum (anti-MBP-Lhc1 or anti-MBP) followed by HRP GAM IgA, IgG and IgM (Sigma) and developed using ECL substrate (SuperSignal, West Pico, Thermo Electron, Waltham MA).

Immunofluorescence microscopy. Brain autopsy samples were obtained from a 30 year old white woman who died of severe and diffuse meningitis due to a cryptococcal infection. Slides were deparaffinized by Xylene and rehydrated through a series of decreasing percentages of ethanol. Slides were rinsed with running tap water followed by 2 washes in TBS plus 0.025% Triton X-100. After blocking in 10% normal serum with 1% BSA in TBS for 2 hours at room temperature, slides were incubated with rabbit anti MBP-LHC serum or rabbit anti-MBP serum (1:100 dilution) respectively in TBS with 1% BSA overnight at 4°C. The following day, slides were rinsed with TBS plus 0.025% Triton X-100 and incubated with Alexa Fluor 594 goat anti-mouse IgG (Invitrogen) in TBS with 1% BSA for one hour at room temperature, followed by 3 washes with PBS. Immunofluorescence images were collected under the Leica DMI6000 B fluorescence microscope.

**Biophysical analysis of DMSO-extracted capsular polysaccharide**

Capsular PS samples from H99 wt, *lhc1∆, and lhc1∆+LHC1* strains were isolated by DMSO extraction, prepared and subjected to light scattering analysis as described [[12](#_ENREF_12)]. Zeta potential (
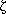
), and shift frequency of polysaccharide samples were calculated using particle electrophoresis in a Zeta potential analyzer (ZetaPlus, Brookhaven Instruments Corp., Holtsville, NY) as described [[13](#_ENREF_13)]. It is derived from the equation
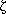
 = (4
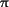

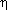
*m*)/*D*, where *D* is the dielectric constant of the medium,
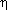
 is the viscosity, and *m* is the electrophoretic mobility of the particle. Polysaccharide molecular mass, radius of gyration, and second virial coefficient values were determined by static light scattering using a differential refractometer and molecular weight analyzer (BI-DNDC, BIMwA, respectively; Brookhaven Instruments, Hotsville, NY, USA). The hydrodynamic size and polydispersity values were determined by dynamic light scattering in a 90Plus/BI-MAS Multi Angle Particle Sizing analyzer (Brookhaven Instruments Corp., Holtsville, NY).

**Cryo-Scanning Electron Microscopy (Cryo-SEM)**

Indicated fungal strains were fixed in 2.5% glutaraldehyde in .1M sodium cacodylate buffer. All specimens were aliquoted into “freeze fracture hats” (Leica Microsystems, Vienna, Austria) for cryoimmobilization in a Leica EMPact2 high pressure freezer (Leica). Hats were transferred into a BAF 060 (Leica) freeze etching device, vacuum at 1x10 -6 mbar and stage temperature at -145°C for fracturing and sputter coating. After fracturing, the specimens were sublimated at -95°C for 15-20 minutes and shadowed at -145° C by electron beam evaporation with 1.8-3.5 nm of platinum at a fixed angle of 45° followed by an additional 14-20 nm of carbon, rotary shadowed at a 90° angle. After coating, frozen samples were mounted in a Gatan CT-3500 cryo-holder (Gatan, Inc, Abingdon, UK) and observed on a Hitachi S-5200 in-lens microscope (Hitachi) at -150°C or colder after 15 min of further sublimation at -95°C within the microscope to remove ice contamination.

**Immunolocalization studies**

Western blots. *C. neoformans* cells were grown in 1:10 Sabaroud media to stationary phase, washed and homogenized by glass bead homogenation [[11](#_ENREF_11)]. Cell wall/capsule and cytosol fractions were separated by centrifugation at 12,000 g x 10 min and cell wall/capsule proteins were extracted from the pellet by a 10 min extraction with SDS buffer (50 mM Tris HCl, pH 8.0, 0.1 mM EDTA, 2% SDS, 10 mM DTT) and clarified by centrifugation at 12,000 *g* for 5 min. 20 mg aliquots of protein were subjected to SDS/PAGE-western blot using 1:500 of the primary serum (anti-MBP-Lhc1 or anti-MBP) followed by HRP GAM IgA, IgG and IgM (Sigma) and developed using ECL substrate (SuperSignal, West Pico, Thermo Electron, Waltham MA).

Immunofluorescence microscopy. Fig. 2E: Frozen sections of capsule induced *C. neoformans* cells were embedded in resin, fixed in cold methanol for 10 min, washed in PBS 3x, blocked with 1% BSA and 1% goat serum and incubated with 1:100 dilution of mouse serum (anti-MBP-Lhc1 or anti-MBP) for 1 h at room temperature followed by secondary FITC-labeled goat anti-mouse IgG, IgM and IgA and Uvitex 2B, washed 3x with PBS, mounted in 50% glycerol and PBS with 0.1 M N-propyl gallate and observed using a Zeiss Axioskop microscope with a 63x objective and a Hamamatsu camera using Axio Vision 4.6 software (Zeiss). Fig. 4A: Capsule induced *C. neoformans* cells were fixed in 4% paraformaldehyde for 10 min, washed in PBS 3x, blocked with 1% BSA for 30 min and incubated with 1:100 diluted Antibody (18B7, 13F1, 12A1or 2D10) for overnight at 4°C followed by 1:1,000 diluted secondary FITC-labeled goat anti-mouse IgG (abcam) for 18B7 or IgM (Invitrogen) for 13F1, 12A1and 2D10, washed 3x with PBS, mounted in ProLong Gold antifade reagent (Invitrogen) and observed using a Leica DMI 6000B microscope with a Hamamatsu camera using LAS AF6000 ver 2.1.2 software (Leica).

**Phagocytosis assay**

Approximately 5 x 10^4^ J774.16 macrophage-like cells/well were plated in complete Dulbecco’s Modified Eagle Medium (DMEM), and incubated overnight at 37°C in a 5% CO_2_ atmosphere. Cells were continuously kept under the stimulation of recombinant murine g-interferon (IFN-g) 100 U ml-1. *C. neoformans* H99 cells grown for 48 h in YPD were washed with PBS (3x), and resuspended in DMEM supplemented with 20% mouse sera (Pel-Freez Biologicals, Rogers, AR, USA), incubated for 20 min in the indicated antibody and subsequently added to macrophage monolayer in 1:5 (macrophage: yeast) ratio. Unopsonized yeast cells resuspended in DMEM (lacking mouse sera) were used as control. After a 1 h incubation at 37°C the macrophage monolayer was washed three times with PBS, and stained with FUN-1 in distilled water. The phagocytosis index was determined by microscopic examination of the number of fungal cells ingested or adherent divided by the number of total macrophages. For each condition, at least 300 macrophage cells were analyzed and were the results from three independent experiments. For analysis of serum-opsonized phagocytosis, *C. neoformans* H99 cells were grown in Sabouraud’s media for 48 h (target cells), washed 3x with PBS and resuspended in DMEM supplemented with 20% C57/BL6 mouse sera, incubated for 20 min and subsequently added to macrophages resulting in a final 1:5 (macrophage : yeast) ratio.

**Fungal killing assay by human monocytes**

The method of Miller and Mitchell for the killing of *C. neoformans* by human monocytes was used [[14](#_ENREF_14)]. Briefly, human peripheral blood was obtained from a normal volunteer with a normal CH50, anti-coagulated with 10 units/ml heparin. Mononuclear cells were purified by using Ficoll-Paque^TM^ density gradient centrifugation. Monocytes were washed twice in PBS containing 0.1% bovine serum albumin, counted, and suspended in RPMI 1640 with 10% Fetal Bovine Serum at the desired concentration. One hundred microliters of effector cells at 3 x 10^6^ viable cells per ml was added to the flat-bottom 96 wells of microtiter plates. The plates were incubated at 37°C under 5% CO_2_ for 2 hours, and the medium was removed and replaced with 100 µl of RPMI with 10% normal human serum. An E:T ratio of 1:1 was achieved by adding 100 µl of yeast cells at 3 × 10^6^ CFU/ml of RPMI-normal human serum. After incubation at 37°C in 5% CO_2_ for 4 h, 100 µl of 0.05% SDS was added to all wells to lyse the monocytes and release phagocytized yeast cells. The wells were washed three times with sterile water, the washes were pooled and serially diluted, and portions of the final dilution (100 µl to 1.0 ml) were placed in sterile petri plates. After 3 days of incubation at 37°C, the CFU of C. *neoformans* were enumerated and the percentage of killing was calculated as follows: (1 - mean experimental CFU/mean control CFU) x 100. The mean CFU of each experimental and control value was calculated from five plates as described previously [[14](#_ENREF_14)].

**Fungal killing by J774A.1 cells**

The method of Wormley and Perfect was used [[15](#_ENREF_15)]. Briefly, 1x10^5^ cells of J774A.1 (obtained from the American Type Culture Collection) in a volume of 50 μl per well was added to flat-bottom 96 wells plates and supplemented with interferon-γ (100 U/ml) and lipopolysaccharide (0.6 μg/ml) and incubated at 37°C with 5% CO_2_ for 12–18 h. An overnight culture of the indicated strains of *C. neoformans* were washed at 900*g* 3x for 5 min, and yeast pellets were suspended in 10 mL of DMEM culture media and the number of viable yeast cells quantified. Yeast cell suspensions at 10^6^ cells per mL were suspended in DMEM culture media containing mAb18B7 (1 μg/ml) and incubated for 1 h at 37°C with 5% CO_2_. Yeast cells were added (10^5^ cells/100 μl) to macrophages in 96-well tissue culture plate and incubated at 37°C at 5% CO_2_ for 1 h then washed 3x with sterile phosphate-buffered saline to remove extracellular yeast. After the removal of extracellular yeast, 200 μl of DMEM culture media were added to each well and again incubated at 37°C for 81 h. 100 µl of  0.05% SDS was added to all wells to lyse monocytes and release phagocytized yeast cells. The wells were washed three times with sterile water, the washes pooled and serially diluted, and portions of the final dilution (100 µl to 1.0 ml) were quantified by CFU.

**Mouse virulence model with the addition of cobra venom factor**

The method of Shapiro *et al* [[16](#_ENREF_16)] was used. Briefly, CVF was injected into mice one day prior to *Cryptococcus* infection. Five units (5 U) of CVF (Quidel, San Diego, CA), diluted in 100 µl of sterile, pharmaceutical-grade physiological saline, was injected IP twice, 4 h apart. Four days later, mice received another single injection of CVF at the same dose, route, and volume. Blood was sampled at pre and post injection and C3 assayed by ELISA that demonstrated that at least 80% of serum complement was depleted from serum.

**Detection of C3 and iC3b binding by flow cytometry**

Sixty microliters of *Cryptococcus* (1× 10^7^ CFU/ml) was incubated with 40 µl serum in the presence of 1 mM MgCl_2_ and 0.15 mM CaCl_2_. To inhibit the classical and lectin pathways, EGTA was added to a final concentration of 40 mM in the presence of 5 mM MgCl_2_. After incubation at 37°C for 30 min, the reaction was stopped with 1 ml ice cold 10 mM EDTA in PBS. Cells were centrifuged (850 × *g* for 5 min at 4°C) and washed two times with 1 ml PBS. Cells were stained with 50 µl of 1:500 goat anti-mouse C3-FITC (ICN, Aurora, OH) or 10 µg/ml of mouse anti-human C3 (Lifespan Biosciences) and incubated on ice for 30 min. Alternatively, mouse anti-human iC3b (Quidel) was utilized after conjugation to PE using the Zenon Mouse IgG labeling kit (Life Technologies) according to the manufacturer’s directions. Cells were washed two times with 1 ml PBS and fixed with 200 µl 2% paraformaldehyde. Cells were then washed two times with 1 ml PBS and resuspended in 300 µl PBS with 1% bovine serum albumin (BSA, Sigma Aldrich, Saint Louis, MO) and 0.02% sodium azide. Flow cytometry was conducted on a Becton Dickinson LSR Fortessa using Cell Quest Pro software. Data were analyzed using FlowJo software (Tree Star, Inc., Ashland, OR).

**Supplemental References:**

1. Zhu X, Williamson PR (2004) Role of laccase in the biology and virulence of Cryptococcus neoformans. FEMS Yeast Res 5: 1-10.

2. Panepinto J, Liu L, Ramos J, Zhu X, Valyi-Nagy T, et al. (2005) The DEAD-box RNA helicase Vad1 regulates multiple virulence-associated genes in *Cryptococcus neoformans*. J Clin Invest 115: 632-641.

3. Erickson T, Liu L, Gueyikian A, Zhu X, Gibbons J, et al. (2001) Multiple virulence factors of *Cryptococcus neoformans* are dependent on *VPH1*. Mol Microbiol 42: 1121-1131.

4. Varma A, Edman JC, Kwon-Chung KJ (1992) Molecular and genetic analysis of *URA5* transformants of *Cryptococcus neoformans*. Infect Immun 60: 1101-1108.

5. Cox GM, Toffaletti DL, Perfect JR (1996) Dominant selection system for use in *Cryptococcus neoformans*. J Med Vet Mycol 34: 385-391.

6. Waterman SR, Park Y-D, Raja M, Qiu J, Hammoud DA, et al. (2012) Role of CTR4 in the Virulence of Cryptococcus neoformans. MBio 3.

7. Liu X, Hu G, Panepinto J, Williamson P (2006) Role of a *VPS41* homolog in starvation response and virulence of *Cryptococcus neoformans*. Mol Microbiol 61: 1132-1146.

8. Bhaumik S, DePuy J, Klimash J (2007) Strategies to minimize background autofluorescence in live mice during noninvasive fluorescence optical imaging. Lab Anim (NY) 36: 40-43.

9. Panepinto J, Komperda K, Frases S, Park Y, Djordjevic J, et al. (2008) Sec6-dependent sorting of fungal extracellular exosomes and laccase of *Cryptococcus neoformans*.

10. Shimizu S, Kataoka M, Shimizu K, Hirakata M, Sakamoto K, et al. (1992) Purification and characterization of a novel lactonohydrolase, catalyzing the hydrolysis of aldonate lactones and aromatic lactones, from Fusarium oxysporum. Eur J Biochem 209: 383-390.

11. Olson GM, Fox DS, Wang P, Alspaugh JA, Buchanan KL (2007) Role of protein O-mannosyltransferase Pmt4 in the morphogenesis and virulence of Cryptococcus neoformans. Eukaryot Cell 6: 222-234.

12. Cordero RJ, Frases S, Guimaraes AJ, Rivera J, Casadevall A (2011) Evidence for branching in cryptococcal capsular polysaccharides and consequences on its biological activity. Mol Microbiol 79: 1101-1117.

13. Nosanchuk JD, Casadevall A (1997) Cellular charge of Cryptococcus neoformans: contributions from the capsular polysaccharide, melanin, and monoclonal antibody binding. Infect Immun 65: 1836-1841.

14. Miller MF, Mitchell TG (1991) Killing of Cryptococcus neoformans strains by human neutrophils and monocytes. Infect Immun 59: 24-28.

15. Wormley FL, Perfect J, editors (2005) Antifungal agents: Methods and protocols. Totowa, NJ: Human Press. 193-198 p.

16. Shapiro S, Beenhouwer DO, Feldmesser M, Taborda C, Carroll MC, et al. (2002) Immunoglobulin G monoclonal antibodies to Cryptococcus neoformans protect mice deficient in complement component C3. Infect Immun 70: 2598-2604.
